# Limited seed retention during winter inhibits vegetation establishment in spring, affecting lateral marsh expansion capacity

**DOI:** 10.1002/ece3.5781

**Published:** 2019-11-04

**Authors:** Marin van Regteren, Irene Colosimo, Pepijn de Vries, Marinka Elisabeth Barbara van Puijenbroek, Victor Sebastiaan Freij, Martin Josephus Baptist, Kelly Elschot

**Affiliations:** ^1^ Wageningen Marine Research Wageningen University and Research Den Helder The Netherlands; ^2^ Faculty of Civil Engineering and Geosciences Delft University of Technology Delft The Netherlands

**Keywords:** *Aster tripolium*, lateral expansion, *Salicornia* spp., salt marsh, sediment dynamics, seed bank, seed viability, *Spartina anglica*

## Abstract

Coastal systems worldwide deliver vital ecosystem services, such as biodiversity, carbon sequestration, and coastal protection. Effectivity of these ecosystem services increases when vegetation is present. Understanding the mechanisms behind vegetation establishment in bio‐geomorphic systems is necessary to understand their ability to recover after erosive events and potential adaptations to climate change. In this study, we examined how seed availability affects vegetation establishment in the salt marsh–intertidal flat transition zone: the area with capacity for lateral marsh expansion. This requires vegetation establishment; therefore, seed availability is essential. In a 6‐month field experiment, we simulated a before and after winter seed dispersal at two locations, the salt‐marsh vegetation edge and the intertidal flat, and studied seed retention, the seed bank, and the seed viability of three pioneer marsh species: *Salicornia procumbens*, *Aster tripolium*, and *Spartina anglica*. During winter storm conditions, all supplied seeds eroded away with the sediment surface layer. After winter, supplied seeds from all three species were retained, mostly at the surface while 9% was bioturbated downwards. In the natural seed bank, *A. tripolium* and *S. anglica* were practically absent while *S. procumbens* occurred more frequently. The viability of *S. procumbens* seeds was highest at the surface, between 80% and 90%. The viability quickly decreased with depth, although viable *S. procumbens* seeds occurred up to 15 cm depth. Only when seeds were supplied after winter, many *S. procumbens* and some *S. anglica* individuals did establish successfully in the transition zone. Viable seed availability formed a vegetation establishment threshold, even with a local seed source. Our results suggest that, although boundary conditions such as elevation, inundation, and weather conditions were appropriate for vegetation establishment in spring, the soil surface in winter can be so dynamic that it limits lateral marsh expansion. These insights can be used for designing effective nature‐based coastal protection.

## Graphical abstract

Germinated seedlings of *Salicornia procumbens* in the transition zone; a first step of lateral salt‐marsh expansion. Seeds were retained at the salt‐marsh edge and tidal flat when dispersed after winter in March, but not when dispersed before winter in December. *Salicornia procumbens* was the only pioneer present in the natural seed bank with viable seeds at depth. Early dispersal of pioneer marsh seeds may limit vegetation establishment in spring.

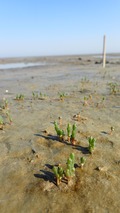

## 1 INTRODUCTION

Coastal areas and estuaries worldwide deliver vital ecosystem services, such as nursery areas for fish, carbon sequestration, food, habitat for unique species, recreation, and flood protection (Harley et al., 2006; McLeod et al., 2011). Nature‐based flood protection is most effective when vegetation is present and it increases with the width of the vegetated area (Barbier et al., 2008; Bouma et al., 2014). Expansion of natural vegetated barriers can occur in the transition zone in front of the system, through the establishment of pioneer vegetation. Due to erosion, pioneer plant cover exhibits cyclic behavior of expansion and retreat (Balke, Stock, Jensen, Bouma, & Kleyer, 2016). This cyclic pattern can be influenced by climate change in bio‐geomorphic systems, such as mangroves (Balke et al., 2013), riparian environments (Karrenberg, Edwards, & Kollmann, 2002), dunes (Huiskes, 1977), and salt marshes (Elsey‐Quirk, Middleton, & Proffitt, 2009; Kirwan, Temmerman, Skeehan, Guntenspergen, & Fagherazzi, 2016). To predict the adaptability of estuarine bio‐geomorphic systems to climate change and ecosystem recovery after erosion, it is necessary to understand the formation and development of these systems (Bouma et al., 2016; Friedrichs & Perry, 2001). The last century, estuaries and coastal areas are suffering from depletion and degradation worldwide (Lotze et al., 2006), and 50% of salt marshes have been lost or degraded (Barbier et al., 2008). Essential requirements for the establishment of pioneer plants and expansion include a suitable habitat, appropriate environmental conditions, and the availability of propagules, often seeds (Erfanzadeh, Garbutt, Petillon, Maelfait, & Hoffmann, 2010; Garbutt, Reading, Wolters, Gray, & Rothery, 2006). Once seeds are produced, their availability will be determined by dispersal, retention, the seed bank, and viability of the seeds (Balke et al., 2011; Chang, Veeneklaas, Buitenwerf, Bakker, & Bouma, 2008; Rand, 2000; Wolters, Garbutt, & Bakker, 2005).

In salt marshes of North‐Western Europe, plants generally have ripened seeds in autumn and reported dispersal period range from autumn to winter (Huiskes et al., 1995; Hutchings & Russell, 1989; Rand, 2000; Wolters et al., 2005). Seed dispersal in salt marshes is mostly local (Ellison, 1987; Rand, 2000). The bare intertidal flats in front of the salt marsh may provide suitable areas for colonization of pioneer marsh plants, that is, the initial step of lateral marsh expansion. However, seed availability can potentially form a bottleneck for pioneer vegetation establishment (Friess et al., 2012; Wolters et al., 2005).

Once dispersed to a suitable habitat, seed retention is affected by environmental variables which ultimately control successful plant establishment. Factors shown to influence seed retention and the local seed bank are sedimentation (Balke et al., 2013) and erosion rates (Houwing, 2000; Zhu, Bouma, Ysebaert, Zhang, & Herman, 2014), topographic heterogeneity (Chang et al., 2008), and bioturbating infauna (Delefosse & Kristensen, 2012). The burial of seeds, through sedimentation or bioturbation, can increase seed retention, as they are protected against erosive forces under a layer of sediment (Zhu et al., 2014). However, these seeds may end up buried too deep in the soil (Erfanzadeh, Garbutt, et al., 2010), disabling their ability to meet their light requirement (Ungar & Riehl, 1980) or their ability to reach the soil surface after sprouting in spring (Jurik, Wang, & Vandervalk, 1994). The availability of seeds closer to the surface is heavily reduced by erosion of the upper soil surface layer (Houwing, 2000). The seeds at the surface are transported away by currents and waves (Groenendijk, 1986; Huiskes et al., 1995). Both the temporal and spatial differences in sediment dynamics in the field and their effect on the preseedling stage of salt‐marsh pioneers remain under‐studied at present.

It is possible that the retention of seeds in the soil may result in a persistent seed bank. Although seed banks have been recognized as key for the success of restoration and conservation efforts, research on riverine, estuarine, and marine seed banks and related processes has remained sparse (Goodson, Gurnell, Angold, & Morrissey, 2001). Currently, it is uncertain how dependent salt marshes are on a seed bank for lateral expansion (Bossuyt & Honnay, 2008; Erfanzadeh, Garbutt, et al., 2010). Furthermore, a seed bank can only advance lateral expansion when seeds remain viable. Studies on viable seed availability in areas where vegetation expansion is prone to occur, such as the salt marsh to the intertidal flat transition zone, are scarce. A seed bank was found to be important for the annual *Salicornia europaea* in years with low seed abundance (Ungar, 1987), but this plant species occurs higher up the marsh (Davy, Bishop, & Costa, 2001). The seed bank and its viability in the marsh transition zone remain to be examined on a deeper level.

The objective of this study is to examine whether seed availability can form a threshold for vegetation establishment in the transition zone even with a source population in proximity. These issues were addressed in a Wadden Sea salt marsh which has been naturally accreting and expanding the past three decades (Baptist, Vroom, et al., 2019). Three frequently occurring species with discriminant seed size and growing seasons were used to account for variable life histories. In order to gain insight into potential lateral expansion of salt marshes in the transition zone, the specific aims were to assess (a) how seed retention is affected by dispersal time, before winter and after winter; (b) the abundance of seeds in the naturally present seed bank; (c) the viability of seeds retrieved from different depths; and (d) vegetation establishment success in the transition zone.

## 2 METHODS

### 2.1 Study area

The salt marsh at Zwarte Haan, The Netherlands (53°18′36N, 5°37′08E), was used as a model system. It is a naturally developing salt marsh in the Wadden Sea located along the Dutch main coast. Current habitat conditions, such as elevation, hydrodynamic regime, and presence of a source population, are appropriate for the establishment of pioneer vegetation in the transition zone (Balke, Herman, & Bouma, 2014; Rand, 2000; Wang & Temmerman, 2013), giving us the opportunity to study essential processes that affect initial vegetation establishment. This tidal marsh is dominated by plants typical for pioneer, low, and high marshes (Petersen, Kers, & Stock, 2014). The focus lies on the pioneer species and their ability to colonize the preceding transition zone. Many of the pioneer species are annuals that reproduce by seed, *Salicornia* spp., *Suaeda maritima*, and *Atriplex portulacoides*. *Aster tripolium* is a biennial that also reproduces by seeds. *Spartina anglica* is a pioneer species that can spread vegetatively and through rhizomes as well as reproduce with seeds. In brackish marshes, *Scirpus maritimus* is a pioneer species that can spread vegetatively, through rhizomes and with seeds (Silinski et al., 2016). This pioneer zone is mostly dominated by the annual *Salicornia procumbens* (glasswort), patches of the perennial *S. anglica* (common cordgrass), and individuals of the biennial *A. tripolium* (sea aster) (Table 1). As in other marshes (Egan & Ungar, 2000), seed‐reproducing species are dominant in this pioneer zone. The astronomical tidal range is approximately 3 m during spring tide and 1.5 m during neap tide, and the elevation at the study site was approximately 1 m above NAP (Dutch Ordnance Datum). The mud content (<63 μm) of the sediment was around 68%. The bathymetry contained a gentle slope of 0.0035‰. On the nearby intertidal flat, Westhoek, located approximately 10 km southwest from our field site, sediment dynamics were extensively monitored from December 2017 until February 2018.

**Table 1:** Seed characteristics, based on this study combined with data adapted from Rand (2000) and Wolters et al. (2004)

Species	Shape	Size (mm)	Surface	Dispersal
*Salicornia procumbens*	Oval	1–2	Hairs	Water
*Aster tripolium*	Elongated	5–7	Hairs, plume	Wind
*Spartina anglica*	Elongated	15–20	Smooth	Water

### 2.2 Seed‐sediment mixture

To simulate seed dispersal, a seed‐sediment mixture was created for the manual seed additions. This mixture consisted of sediment with a surplus of seeds from *S. procumbens*, *A. tripolium*, and *S. anglica*. Seeds were collected in autumn 2017 to ensure that they were ripe, though not yet dispersed. *Salicornia procumbens* and *A. tripolium* seeds were collected in the pioneer zone of Westhoek (53°25′03N, 6°22′49E). Dead standing biomass of *S. procumbens* containing seeds was cut into smaller branches. Additionally, individual seeds were added to the seed mixture, as *S. procumbens* is known to spread with branches holding seeds and individual seeds (Smit van der Waaij, Houwing, Duin, Dijkema, & Smit, 1995). Flower heads containing *A. tripolium* seeds were gathered at Westhoek. *Spartina anglica* seeds were collected at the Balgzand salt marsh (52°53′56N, 4°50′46E, The Netherlands) in October 2017, dried, and loosened from their spike to obtain individual spikelets, mimicking its dispersal strategy. The plants were dried in a climate chamber with continuous ventilation for 14 days. Approximately 40 L of sediment, 68% mud (<63 μm), was collected at the study site. A surplus of seeds was homogenously mixed into the sediment and divided into forty even portions.

### 2.3 Experimental design

**Figure 1:**
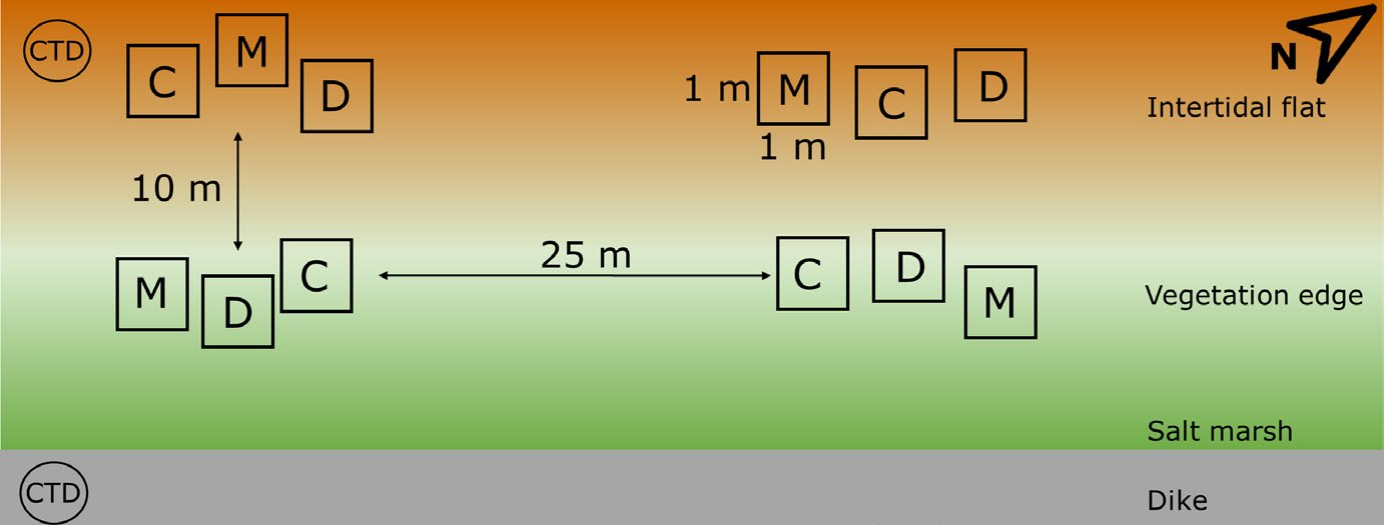
The experimental design of the field experiment. Two of the ten replicate blocks are displayed. Each block consisted of two locations: the intertidal flat and the vegetation edge. Each zone contained three 1 × 1 m plots corresponding to control (C), December (D), and March (M) seed dispersal treatments. Three sediment cores were taken from each plot. One CTD‐diver measured water levels for the duration of the experiment; a second CTD‐diver located on the dyke in permanent dry conditions was used to correct for air‐pressure variability

The field experiment lasted from December 2017 through May 2018. Two zones were included, the vegetation edge of the marsh pioneer zone, identified by the presence of dead standing material of *S. procumbens*, and the bare intertidal flat 10 m seawards in front of the vegetation edge, to assess expansion potential. Each zone consisted of 10 replicate blocks with approximately 25 m between them, each containing three 1 × 1 m experimental plots (Figure 1). These consisted of a control plot and two seed treatment plots. The seed treatments consisted of two temporally distinct manual seed additions, to simulate different dispersal times. The first seed addition was conducted before winter, December 2017, and the second seed addition was executed after winter, March 2018. A control treatment was included to examine natural seed availability. The treatments will hereafter be referred to as control (no seed addition), December (December seed addition), and March (March seed addition). Twenty seed mixtures were applied in a thin layer (±1 mm thick) on the surface of 10 plots at the vegetation edge and 10 plots on the intertidal flat in December 2017. The remaining 20 mixtures were stored in a dark and cool environment (3°C) until the seed addition in March 2018. No seeds were added to the 20 control plots. Final sediment cores were taken in May 2018 to assess seed depth distribution.

### 2.4 Seed retention, seed bank, and viability of seeds

To assess seed retention and the seed bank, in May 2018, three randomly placed sediment cores with 5.8 cm diameter were taken, up to 15 cm deep in each plot. The three sediment cores together covered a total surface area of 80 cm^2^. This resulted in 180 sediment cores in total, 60 cores for each treatment (control, December and March). Of these 60 cores, 30 cores were collected at the vegetation edge and 30 cores were collected at the intertidal flat to estimate location effects.

The top 5 cm of the soil was sliced into layers of 1 cm, and the next 10 cm (5–15 cm deep) of soil was sliced into layers of 2 cm thick. This resulted in the following layers: 0–1, 1–2, 2–3, 3–4, 4–5, 5–7, 7–9, 9–11, 11–13, and 13–15 cm. In the statistical analysis, the center of each layer is used to represent the depth. Each sediment layer was sieved over a 500 µm mesh to filter out all seeds from the sediment. Everything larger than 500 µm was transferred to a transparent gridded tray, placed on a light table, and analyzed for seeds. For the surface layer (0–1 cm), the number of seedlings for each of the three plant species was recorded. In each succeeding layer, the number of seeds for each of the three plant species was recorded. Thereafter, all seeds were transferred to the climate chamber for germination to determine seed viability, following the germination protocol by Ter Heerdt, Verweij, Bekker, and Bakker (1996).

### 2.5 Bed‐level elevation and water‐level monitoring

The surface elevation was measured at the four corners of each experimental plot using a RTK‐DGPS. The averaged elevation of these four measurements was taken as the bed‐level elevation for each plot. The average elevation of all treatment plots at the vegetation edge was 1.00 ± 0.01 m above NAP, and elevation of intertidal flat plots was 0.96 ± 0.01 m above NAP. The elevation measurement was repeated at the beginning of each treatment (i.e., December 2017 and March 2018) and at the end of the entire experiment (May 2018). The elevation of the December treatment plots was additionally measured in December 2017 and March 2018, and the elevation of the March treatment plots was measured in March 2018. One CTD‐diver (Conductivity‐Temperature‐Depth meter, DI27x, Eijkelkamp Soil & Water) was installed to acquire water levels, providing information on the inundation conditions throughout the experiment. Another CTD was placed on top of the dyke to correct for the first CTD for local air‐pressure variability.

At Westhoek, hydrodynamic forcing and bed‐level changes were measured at four locations of the intertidal flat. The most elevated site was located about 400 m seaward of the vegetation edge at an elevation of 0.15 m NAP. The water‐level and bed‐level change at this location is used in this study to show the effect of two storms occurred during the winter experiment. The water level was measured continuously using a pressure sensor (OSSI‐010‐003C, Ocean Sensor Systems) at 10 Hz frequency, installed at 2 cm from the seabed. These pressure data were corrected for the air‐pressure variability using a second CTD. The second CTD was installed at the measurement location, avoiding inundation of the instrument. The bed‐level data were collected using an ADV (Acoustic Doppler Velocimeter, Nortek) in burst mode, a method frequently used to measure sediment erosion and deposition (Andersen, Fredsoe, & Pejrup, 2007) and applicable for tidal marsh research (Nolte, Koppenaal, et al., 2013). It was installed ±20 cm from the seabed and measured the distance between the probe and the seabed at the beginning and end of each 10‐min measurement interval, with an accuracy of ±1 mm. Zwarte Haan and Westhoek are similar regarding bed material, that is, grain size distribution (Boer, 2015) and the bathymetry, for example, the slope of the bed in the intertidal zone and the geographical orientation of the coastline. These conditions suggest a similarity in the exposure to the hydrodynamic forcing. However, given the differences in bed elevation (1 m NAP at Zwarte Haan and 0.15 m NAP at Westhoek), the bed‐level variation at Westhoek was not used to extrapolate the bed‐level variation at Zwarte Haan. The bed‐level changes at Westhoek exemplify the effect of storm conditions on sediment erosion and deposition.

### 2.6 Data analyses

Three replicate sediment cores from each plot were pooled to obtain seed abundance data. For the top 5 cm, abundances were counted per 80 cm^3^; for the lower 10 cm, abundances were counted per 160 cm^3^. For samples between 5 and 15 cm deep, abundances were estimated per 80 cm^3^, by dividing total abundance with the thickness of the layer (2 cm). Viability was calculated based on the fraction of the recovered seeds that germinated successfully in laboratory conditions. This was repeated for all treatment and depth combinations. Samples without seeds present were excluded from the viability analysis.

The elevation of the experimental plots at Zwarte Haan was used to estimate total bed‐level change during and after winter. The high‐resolution bed‐level change at Westhoek was calculated as the difference in distance to the bed from the instrument probe at the beginning and end of each measurement burst (10‐min frequency). The measurements of bed‐level change and water level were then averaged over, respectively, 1‐hr and 10‐min intervals.

The abundance of *A. tripolium* and *S. anglica* seeds in the control and December treatments as well as below 3 cm in the March treatment was insufficient for statistical analyses. The results of *A. tripolium* and *S. anglica* seed abundance will, however, be presented and qualitatively discussed. Analyses on seed viability and depth distribution of the seed bank were performed on *S. procumbens*.

Firstly, seed abundance was analyzed with a generalized additive model (GAM) (Wood, 2006). Generalized Additive Model was selected as the modeling instrument as the effect of depth was not linear. Furthermore, a GAM was applied to smoothen expected autocorrelation between depth layers. The fixed factors were as follows: smoothed depth (interaction with treatment), location, and treatment. Block was considered to be a random effect (Wood, 2008). The smooth function used penalized regression splines with 5 knots, where the number of knots reflects the degrees of freedom (i.e., the flexibility of the curve) required by the spline (Wood, 2008). An offset was used to correct for varying sample thickness at different depths. Because a Poisson distribution resulted in an overdispersed fit, the data were analyzed with a log‐linked negative binomial distribution. To correct for the sampled depth layer thickness, both model outcome and observed counts were eventually expressed in numbers per 80 cm^3^ soil.

Next, viability, expressed as the fraction of successfully germinated seeds/seedlings of the total seeds, was analyzed using a GAM with a binomial distribution with a logit link function. The fixed factors were as follows: smoothed depth (interaction with treatment), location, and treatment. The block was again considered to be a random effect. The smooth function used penalized regression splines with 8 knots. Only sampled depth layers with *S. procumbens* seeds present were used for the viability analysis.

Finally, seedling establishment was analyzed with a generalized least squares (GLS) to allow for unequal variances (heteroscedasticity) (Pinheiro & Bates, 2000). The fixed factors were as follows: species, location, and weighted treatment. Block was considered to be a random effect.

All confidence intervals were plotted as 1.96 × *SE* (i.e., 95% confidence). Likelihood ratio tests (LR) were used to assess the significance of individual factors. Model assumptions were assessed graphically (Zuur & Ieno, 2016). All statistical analyses were performed with the statistical program R (R Core Team, 2015) using additional packages: “ggplot2” for plotting (Wickham, 2009), “nlme” and “lme4” for linear mixed effect models (Bates, Machler, Bolker, & Walker, 2015; Pinheiro, Bates, DebRoy, & Sarkar, 2019), and “mgcv” for GAM models (Wood, 2011).

## 3 RESULTS

### 3.1 Seed retention

**Figure 2:**
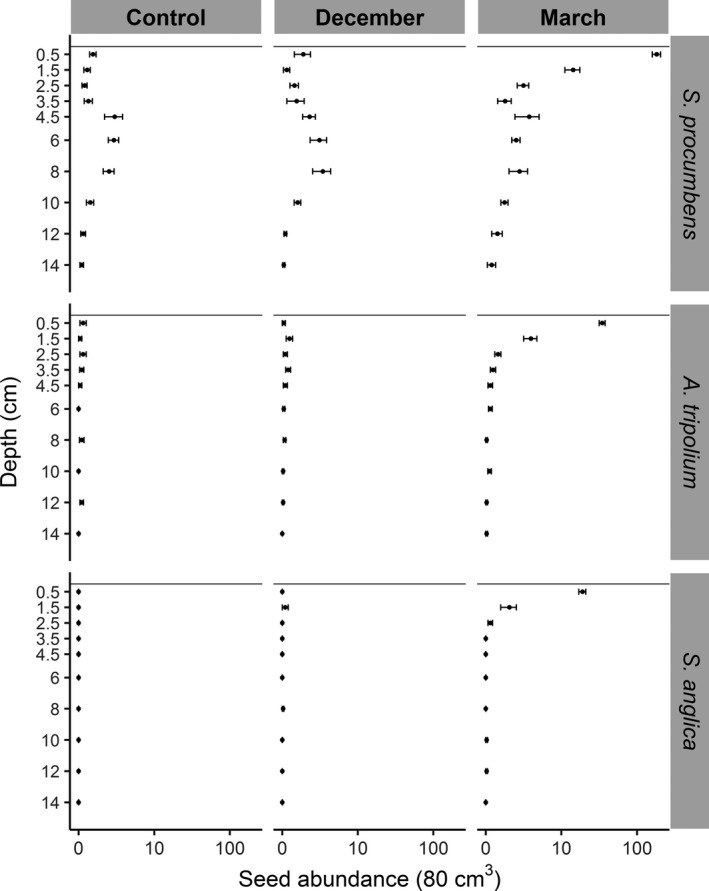
Seed abundance (log‐scale on the horizontal axis) along depth for three seed dispersal treatments (columns): control, December, and March; and three pioneer species (rows): *Salicornia procumbens*, *Aster tripolium*, and *Spartina anglica*. Means with 95% confidence intervals (CI) are indicated; pioneer and intertidal flat data were combined for brevity, *n* = 60

**Figure 3:**
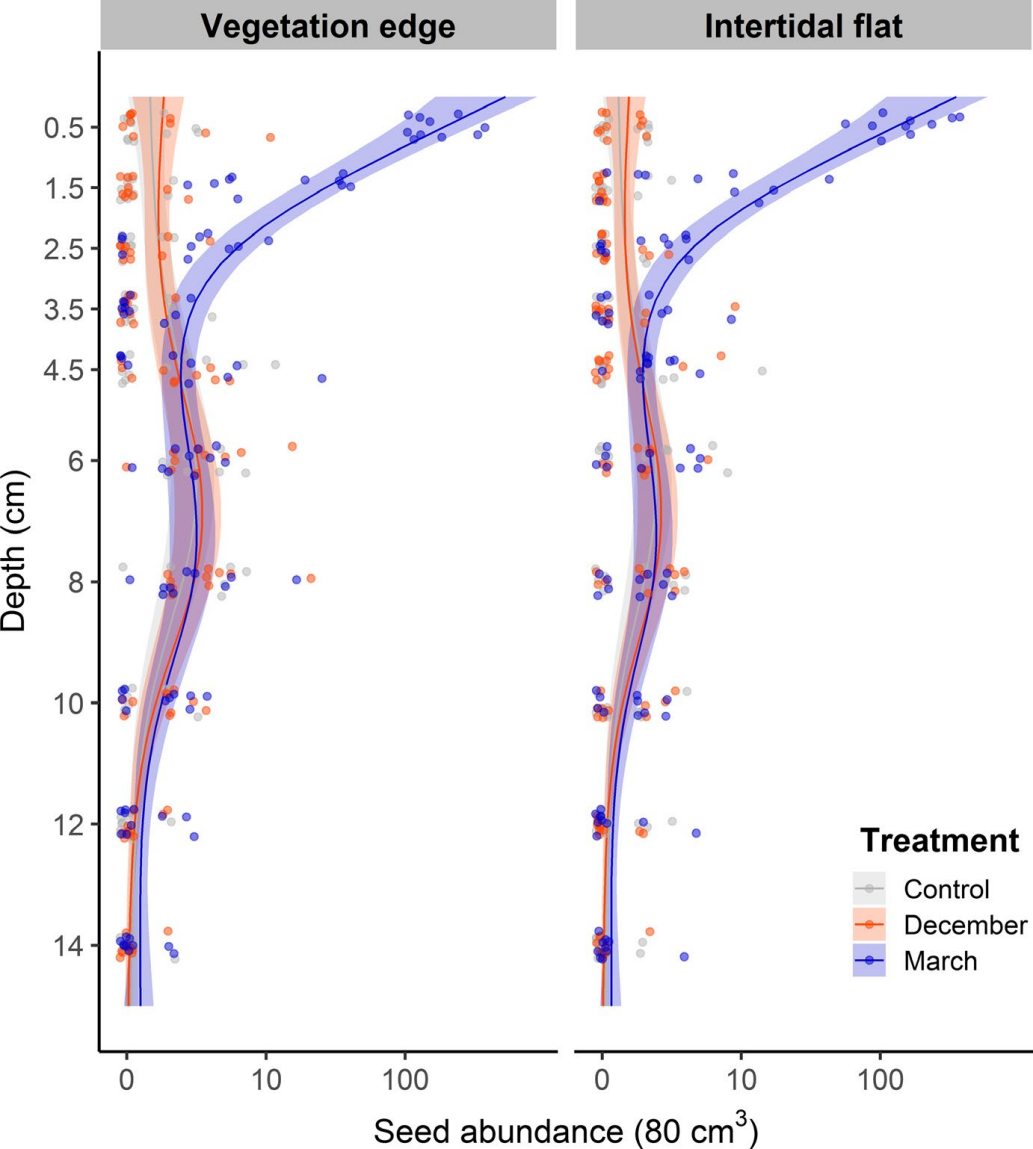
*Salicornia procumbens* seed abundance of the vegetation edge and the intertidal flat with 95% CI for three seed dispersal treatments along depth, as predicted from the GAM model. Jittered dots indicate individual samples (log‐scale on the horizontal axis), *n* = 30

In May, the total abundance of seeds retrieved was similar for the December treatment (272 seeds) and control (233 seeds), while the March treatment was much higher (3,755 seeds). For the March treatment, the highest abundances of seeds for all three species were found in the top 2 cm of the surface layer (Figure 2). Most of these seeds belonged to *S. procumbens* (Figure 2). Retention of *S. procumbens* seeds was affected by dispersal time (Figure 3 and Table 2). Seeds manually added before winter (December treatment) were not retained. Seeds manually added after winter (March treatment) were retained until the following establishment period (May).

**Table 2:** Generalized additive model diagnostics of abundance and viability analyses

Abundance *Salicornia procumbens* seeds		Adj. *R* ^2^ 0.67	
Parametric coefficients	Estimates	*z*‐value	*p*
Intercept	−1.64 ± 0.16	−10.32	2.00 × 10^–16^
December treatment	0.007 ± 0.19	0.034	0.97
March treatment	1.47 ± 0.17	8.68	2.00 × 10^–16^
Location	−0.41 ± 0.13	−3.08	0.002
**Smooth terms**	**e*df***	**Chi sq.**	***p***
Random effect block	4.5	8.7	0.041
s(depth):Control	3.4	48.8	1.41 × 10^–9^
s(depth):December	3.5	49.9	8.07 × 10^–10^
s(depth):March	3.8	376.0	2.00 × 10^–16^

Viability *Salicornia procumbens* seeds		Adj. *R* ^2^ 0.77	
Parametric coefficients	Estimates	*z*‐value	*p*
Intercept	−2.33 ± 0.54	−4.29	1.79 × 10^–5^
December treatment	0.87 ± 0.61	1.43	0.15
March treatment	1.95 ± 0.55	3.51	4.36 × 10^–4^
Location	0.04 ± 0.08	0.48	0.63
**Smooth terms**	**e*df***	**Chi sq.**	***p***
Random effect block	6.9	29.6	1.45 × 10^–5^
s(depth):Control	3.4	34.1	5.41 × 10^–6^
s(depth):December	3.5	32.4	8.60 × 10^–7^
s(depth):March	1.7	211.5	2.00 × 10^–16^

Note

Estimates presented with *SE*, s(depth) indicates the fitting of a smoothing function, and edf are the degrees of freedom used by the smooth.

At the surface, retention of *S. procumbens* seeds was similar within the transition zone. However, deeper in the soil (4–9 cm), natural *S. procumbens* abundance differed between the vegetation edge and the intertidal flat (Figure 3). The average abundance of seeds between 4 and 9 cm was higher at the vegetation edge than at the intertidal flat (Figure 3).

### 3.2 Seed bank

The control treatment provided information on the naturally occurring seed bank. For *S. anglica*, no seed bank was present: nearly all *S. anglica* seeds found came from the March seed‐addition treatment (0 from the control, 3 from December, and 386 from March). In the March treatment, *S. anglica* seeds were most abundant in the surface layers, 0–1 cm (361 seeds), and 1–2 cm layer (21 seeds).

*Aster tripolium* seeds occurred naturally in the transition zone (18 from the control, 21 from December, and 766 from March), and seeds were found up to 13 cm deep. For the March treatment, *A. tripolium* seeds, similar to *S. anglica*, were most abundant in the surface layer (676 seeds) and 1–2 cm (59 seeds) layer.

*Salicornia procumbens* had a higher natural abundance of seeds than *S. anglica* and *A. tripolium* (233 from the control, 279 from December, and 3,755 from March). *Salicornia procumbens* seeds in the March treatment were, like *A. tripolium* and *S. anglica*, most abundant in the surface layer (3,213 seeds) and at 1–2 cm (247 seeds) (Figure 2). In the control as well as in both treatments, *S. procumbens* seeds were found at all depths. The highest natural abundance occurred at depths between 4 and 9 cm. Abundances were slightly but significantly higher at the vegetation edge than on the intertidal flat (Figure 3, Table 2). *Salicornia procumbens* seed abundance was significantly higher in the March treatment in the upper 3 layers (0–3 cm) (Figure 3).

### 3.3 Seed viability

Natural seed abundance for *S. anglica* was low; in the control treatment, *S. anglica* was absent from all depths. In the December treatment, three seeds were found of which one germinated. For the March treatment, 10% of the 386 *S. anglica* seeds were viable. Viability of *A. tripolium* was naturally low, of the seeds found in the control treatment, one out of 18 germinated. In the December treatment, 21 *A. tripolium* seeds were found, of which none germinated. For the March treatment, 6% of the 766 *A. tripolium* seeds were viable.

**Figure 4:**
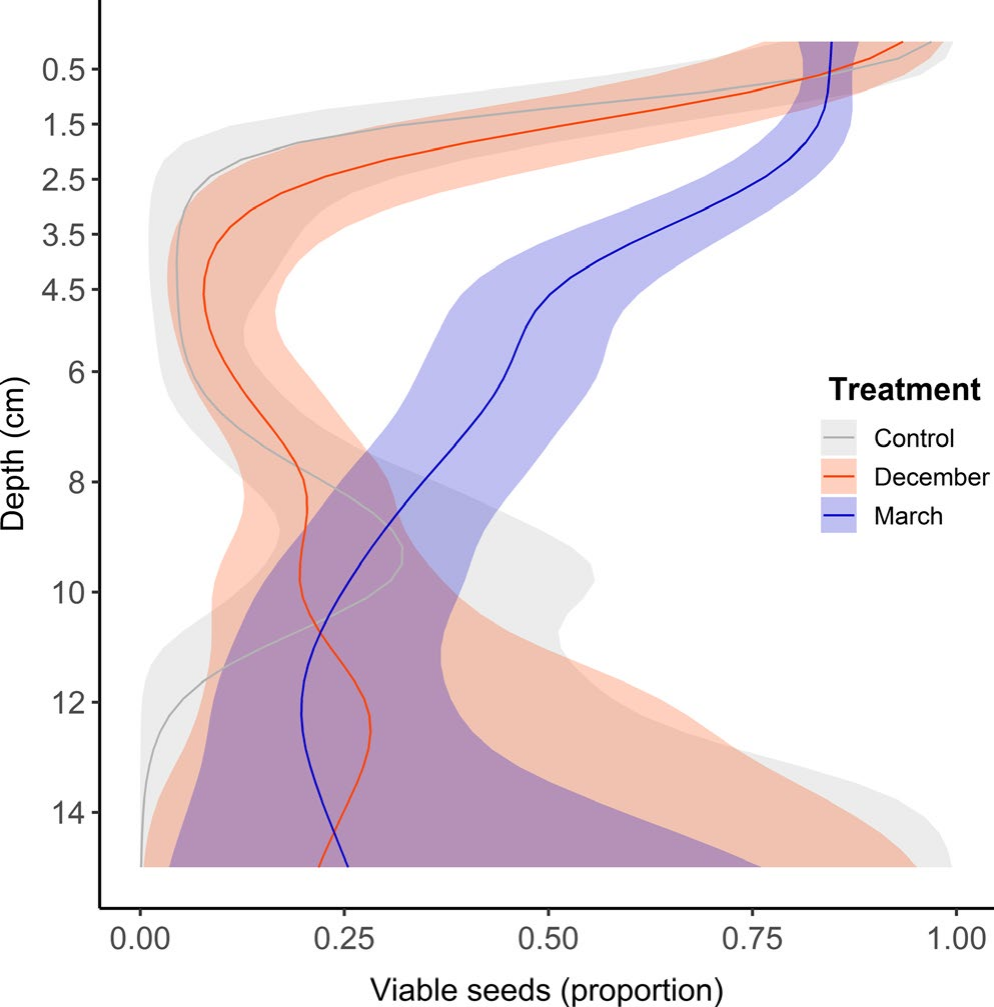
The proportion of viable *Salicornia procumbens* seeds of two seed dispersal treatments and the control along depth with the 95% CI, as predicted from the GAM model

**Figure A1:**
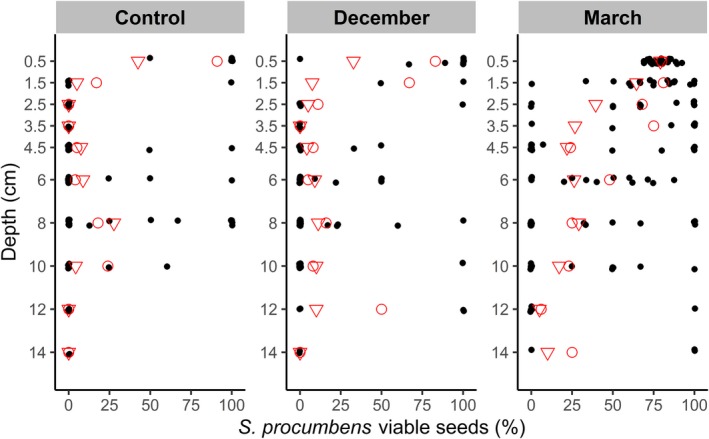
Percentage of viable *Salicornia procumbens* seeds in three treatments over depth. Dots indicate individual samples (277). Red triangles depict the average viability of the samples, and red circles depict the total viability (derived from Table 3)

The viability of *S. procumbens* seeds decreased over depth and differed between treatments (Depth: LR_df=1_ = 255, *p* < .0001; Treatment: LR_df=2_ = 94.15, *p* < .0001, Figure 4 and Figure A1). Seed viability decreased more rapidly with depth in the control and December treatments than in the March treatment (Figure 4). Apart from the surface layer, viability was higher up to 6 cm deep in the March treatment (Figure 4). Viability of seeds was similar at the vegetation edge and the intertidal flat (Table 2). Viability was highest (80% and 90%) in the surface layer (0–1 cm) (Figure 4 and Figure A1). Viable *S. procumbens* seeds were found throughout the 15 cm of soil sampled. In the deepest sampled layers, below 9 cm, the uncertainty around the predicted viability of *S. procumbens* seeds becomes larger due to lower seed abundance (Figure 4, Figure A1 and Table 3).

**Table 3:** The total number of seeds found in every depth × treatment combination, the number of germinated seeds, and the number of samples that contained seeds (max 60)

cm depth	Control			December			March		
	Total seeds	Germinated seeds	In *n* samples	Total seeds	Germinated seeds	In *n* samples	Total seeds	Germinated seeds	In *n* samples
0–1	11	10	10	18	15	10	3,213	2,569	60
1–2	6	1	5	3	2	2	247	199	36
2–3	4	0	4	9	1	6	37	25	18
3–4	7	0	6	11	0	4	16	12	9
4–5	40	2	12	26	2	13	54	13	18
5–7	77	3	27	84	6	25	61	29	23
7–9	61	11	23	98	16	28	71	18	26
9–11	17	4	11	24	2	15	31	7	18
11–13	6	0	4	4	2	4	17	1	8
13–15	4	0	3	2	0	2	8	2	3

### 3.4 Germination of seedlings

**Figure 5:**
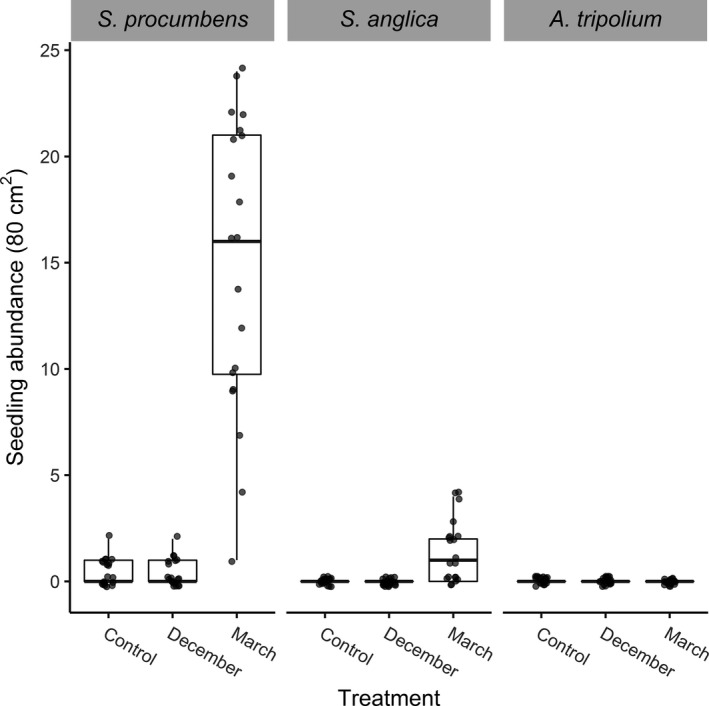
The abundance of established seedlings for three pioneer salt‐marsh species, for control, December, and March treatments. Boxplots with individual plots depicted as dots, *n* = 60. Vegetation edge and intertidal flat data were combined since seedling establishment was similar throughout the transition zone

When sampling in May, seedlings had established in the field (Figure 5). The abundance of seedlings was significantly affected by both treatment and species (GLS, Treatment: LR_df=2_ = 23.9, *p* < .0001; Species: LR_df=2_ = 42.0, *p* < .0001). There were no *A. tripolium* seedlings in the field. *Spartina anglica* seedlings were present solely in the March treatment (Figure 5). *Salicornia procumbens* seedlings were present in similar abundances in the control and December treatment, while the number of germinated *S. procumbens* seedlings was significantly higher in March treatment (Figure 5). The relative proportion of seeds added to the experimental plots was 15:5:1 for, respectively, *S. procumbens*, *A. tripolium*, and *S. anglica*. Location (vegetation edge vs. intertidal flat) had no significant effect on the number of established seedlings (GLS, Location: LR_df=1_ = 0.0008, *p* = .98).

### 3.5 Bed‐level elevation and water level

The RTK‐DGPS measurements showed that on average, the elevation of the experimental plots eroded with 10.9 ± 6.1 mm, from December to March, whereas the plots experienced slight sedimentation 4.4 ± 13.4 mm, from March to May. The erosion of ±10 mm at each plot resulted in the loss of seeds supplied in the December treatment, as the seed‐sediment mixture was 1 mm thick.

**Figure 6:**
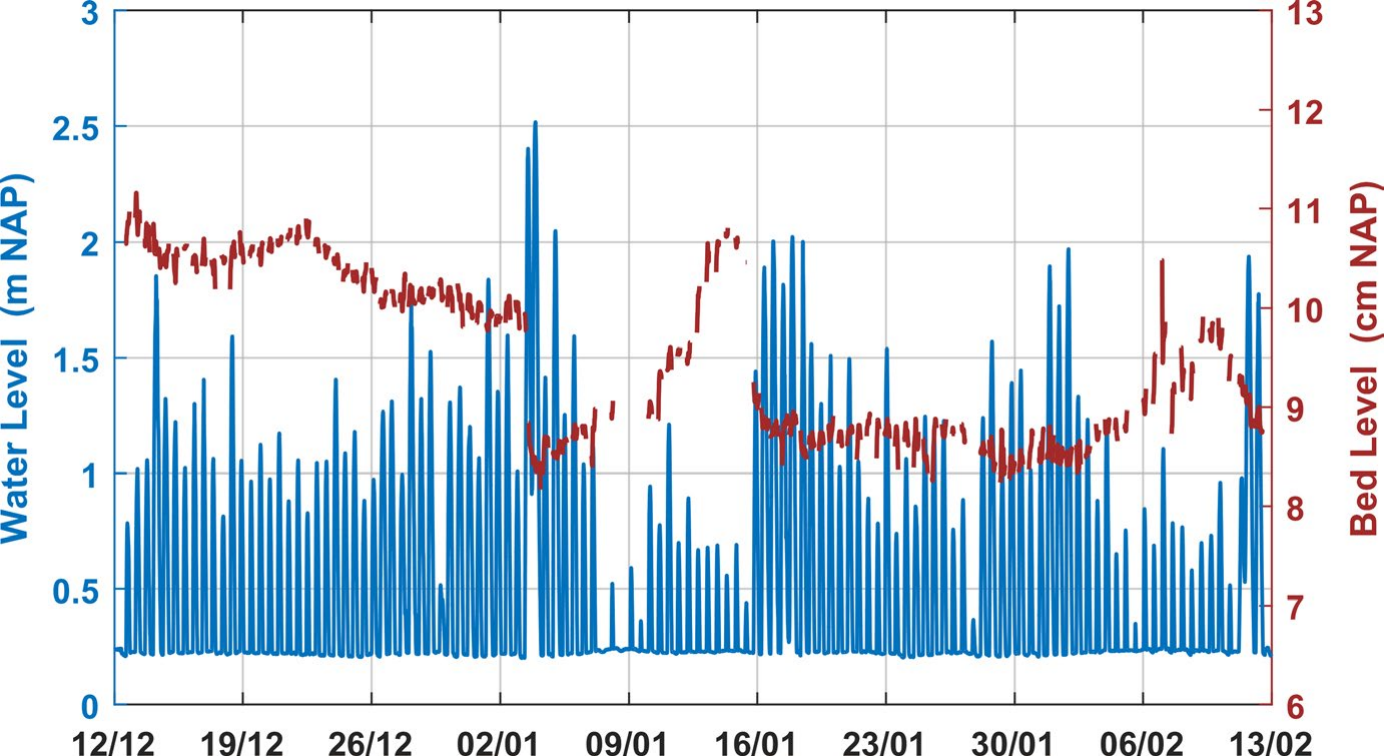
The water level (blue) and the relative bed level (brown) from the start of the experiment until February 13. Two storm events with high water levels in January (3rd and 16th) caused sediment surface erosion. A calm period in between storms resulted in sediment accretion

Information about bed‐level variation during the experiment could not be derived from bed‐level elevation measured at the experimental plots at the start and end of the experiment. To elucidate on bed‐level variation, high‐resolution measurements of the Westhoek tidal flat were used. On January 3rd, a storm with wind speeds exceeding 10 m/s from the southwest generated waves with significant wave height up to 1 m. This resulted in a substantial water‐level setup, with a high water level of 2.5 m NAP (i.e., 1.5 m setup). The storm lasted for 24 hr, that is, two tidal cycles. The water level during low tide was 1 m NAP (coinciding with the mean high tide level of this zone), meaning that the experimental plots, not usually submerged extended periods, were continuously inundated for 24 hr. Exemplifying the consequences of such a storm, the bed‐level change and water‐level data obtained at Westhoek intertidal flat are shown in Figure 6. Higher water levels occurred due to wind‐induced water‐level setup, resulting in bed erosion. This storm was followed by 2 weeks of calm weather, which coincided with neap tide. These conditions are favorable for sediment accretion (Figure 6). The fresh material deposited during this period was easily eroded during a second storm (January 16th, Figure 6).

## 4 DISCUSSION

### 4.1 Seed retention: temporal and spatial patterns

One of our aims was to assess seed retention with different simulated dispersal times in the transition zone. Salt‐marsh pioneer species generally ripen and disperse their seeds in autumn–winter (September to December) in the Northern hemisphere (Huiskes et al., 1995; Hutchings & Russell, 1989; Rand, 2000; Wolters et al., 2005). However, each species has its own dispersal window. *Spartina anglica* has peak seed dispersal times in October and December (Huiskes et al., 1995). *Spartina anglica* seeds still disperse up to march, with the arrival of seeds primarily in January (Zhu et al., 2014). *Salicornia* spp. seeds disperse from October to December, although the months January to spring were not measured (Huiskes et al., 1995; Hutchings & Russell, 1989; Wolters et al., 2005). *Aster tripolium* disperses from September to December (Huiskes et al., 1995; Hutchings & Russell, 1989; Wolters et al., 2005). Other common marsh species, such as *S. maritimus*, *S. maritima*, *Atriplex* spp., and *Puccinellia maritima*, mainly disperse their seeds between September and October (Espinar, Thompson, & Garcia, 2005; Huiskes et al., 1995; Hutchings & Russell, 1989; Rand, 2000; Wolters et al., 2004). On higher elevated marshes, storms have been identified to have a significant influence on seed rain, with year to year variation in peaks, varying from March to December (Chang, Veeneklaas, & Bakker, 2007). Summarizing, seed dispersal mainly occurs between September and December but most studies do not monitor after December (exceptions: Zhu et al., 2014 who look at *S. anglica* specifically and Chang et al., 2007 who focused on the inner marsh). This is an important knowledge gap in salt‐marsh research. We showed that dispersal time is crucial in seed retention. Seeds present in December suffered seed loss due to sediment erosion, but seeds, dispersed after winter, were retained (Figure 2). Houwing (2000) suggested that such a seed loss occurs with a high sand/mud ratio, because candy sediments have a lower erosion threshold and therefore seeds disperse away more easily together with the sediment (Houwing, 1999; Houwing, Duin, Waaij, Dijkema, & Terwindt, 1999). Our study area has a high mud content, 68%, and a loss of seeds due to soil surface erosion was still found during winter; thus, grain size alone cannot explain the loss of seeds. Although the three species studied each have different seed size and characteristics, the retention patterns were similar for all (Figure 2 and Table 1). This implies that also seeds of other marsh species are likely to suffer from the bed elevation sediment dynamics in transition zones.

At Westhoek intertidal flat, spring tides in combination with high wind speeds caused sediment erosion of the upper 1.5–2 cm of the bed (Figure 6). The calm period that followed the erosion event resulted in sediment deposition, indicating that the cumulative bed‐level change in the period was much higher. Although we cannot predict the magnitude of the bed‐level change at Zwarte Haan, we expect that a similar dynamic occurred at this tidal flat, with erosion following from storm conditions and sedimentation occurring in between storms. Multiple storms occurred during the winter months which are likely to cause sediment erosion of the seabed around mean high water, that is, our experimental location (Friedrichs, 2011; Janssen‐Stelder, 2000). At the experimental site (±1 m NAP), CTD data showed that water level reached 1.5 m above the sediment surface. Waves of 1 m on 1.5 m water depth cause wave breaking and thus erosion (Friedrichs, 2011). The storm of January 3rd lasted for 24 hr, that is, two tidal cycles, implying an unusual submergence duration of the site which may result in a decrease in critical shear stress for erosion (I. Colosimo et al., submitted to JGR‐Earth Surface). We, therefore, expect that the bed‐level variation was larger than the 1 cm erosion measured with the RTK‐DGPS during winter. Currents can lead to seed burial and thus to retention as shown in flume studies (Zhu, Cozzoli, et al., 2016); however, in combination with tides and waves it can also lead to seed loss (this study). A dynamic regime can significantly affect seeds transport, that is, burial and loss of seeds, and therefore seed retention and seed bank formation. Additionally, in dynamic areas, measuring elevation before and after experiments may not suffice as bed‐level variation in between could be much greater than the net difference in bed‐level elevation. The sediment fluxes in these shallow intertidal areas are significantly influenced by wind speed and direction, even in moderate wind conditions (I. Colosimo et al., submitted to JGR‐Earth Surface). Consequently, if plants have peak seed dispersal before winter, the risk of seed loss increases. Subsequently, low availability of seeds in the consecutive spring thus limits vegetation establishment. Zhu et al. (2014) show dispersal of *S. anglica* up until march, which may form an adaptation for seed persistence in dynamic systems. Further studies on seed dispersal and retention in the transition zone are still lacking but would greatly contribute to salt‐marsh restoration and conservation purposes.

Depth was a significant factor in seed retention and vertical seed distribution. Seed retention was similar at the vegetation edge and intertidal flat plots. For the March treatment, most supplied seeds, of all species, were present in the surface layer of the sediment (Figure 2). Also in the 1–2 cm layer (March), *S. anglica* and *A. tripolium* seeds were present in significantly higher abundances than in the control. *Salicornia procumbens* was present, up to 3 cm deep (March) in significantly higher abundances than the control (Figure 3). We hypothesize that downward transport of these seeds into the soil occurred through benthic bioturbation (van Regteren, Boer, Meesters, & Groot, 2017; Zhu, Belzen, et al., 2016). Burial of seeds through bioturbation may increase seed retention and create a mechanism for seed bank formation. Superficially buried seeds may aid vegetation establishment in dynamic areas (Goodson et al., 2001; Ungar, 1987; Zhu et al., 2014).

### 4.2 Seed bank

Because seed banks are a key component in restoration and conservation efforts (Goodson et al., 2001), we investigated the naturally present seed bank in the transition zone. This was remarkably different for the three species. *Spartina anglica* was nearly absent (three seeds in total) from the entire transition zone. A likely culprit is the fungus *Claviceps purperea*, which invades the spikelets and impairs the development of *S. anglica* seeds (Boestfleisch et al., 2015). Additionally, the longevity of *S. anglica* seeds is known to be generally low (Wolters & Bakker, 2002), while floating times are relatively long (days) (Koutstaal, Markusse, & De Munck, 1987). Natural dispersal of *S. anglica* seeds may, therefore, be away from the transition zone. This is corroborated by Zhu et al. (2014), that state *S. anglica* to be dependent on seeds for long‐distance dispersal. *Aster tripolium* seeds were sparse in both pioneer and intertidal flat zone. A greater dispersal range is expected because *A. tripolium* is mainly wind‐dispersed (Rand, 2000). *Aster tripolium* can produce many flowers and thus seeds per plant (Nolte, Esselink, & Bakker, 2013), which may compensate for its low viability.

*Salicornia procumbens* seeds were present throughout the entire 15 cm of soil. Remarkably, there was an increase in seed abundance between 4 and 9 cm subsurface. This was consistent in all treatments. These seeds may have been retained and added to the natural seed bank through burial in previous successful years by sedimentation or bioturbation. Additionally, *S. procumbens* seeds occurred in slightly (but significantly) higher abundances at 4–9 cm deep in the vegetation edge than on the intertidal flat. This location effect was not visible in the upper or deeper layers of the soil. The increased seed abundance at the vegetation edge may have originated in years where high reproduction success coincided with local dispersal and less secondary dispersal. So we can conclude that a seed bank was present for *S. procumbens*, the most important pioneer species in the transition zone. Such a seed bank may be more crucial in dynamic areas, where it influences the local establishment success of the species (Ungar, 1987). However, these *S. procumbens* seeds do not germinate when buried below 1 cm (Huiskes, Stienstra, Koutstaal, Markusse, & Soelen, 1985). Thus, for *S. procumbens* to benefit from the seed bank for vegetation establishment, they must occur in the upper layer or come to the sediment surface by sediment erosion or crack propagation (Burmeier, Eckstein, Otte, & Donath, 2010).

Seed banks in transitions zones are not often sampled. It would aid our understanding of vegetation retreat or expansion if future research would focus on the study of the available seed bank and its viability in transition zones. Experimental seed additions in these areas will reveal if vegetation establishment is limited by windows of opportunity (Balke et al., 2011) or the availability of seeds (Erfanzadeh, Garbutt, et al., 2010; Ma, Zhou, & Du, 2011).

### 4.3 Seed viability

To determine the expansion potential of the salt marsh into the transition zone, we examined the seed viability of both naturally occurring and supplied seeds. For *S. anglica* and *A. tripolium*, viability estimation for naturally occurring seeds was impossible, because abundance was too low. The March treatment did contain sufficient seeds, showing that for *S. anglica*, overall viability was 10%. This corresponds to estimates found in literature stating that many *S. anglica* spikelets are not filled and therefore cannot germinate and that the presence of the fungus *C. purperea* lowers viability (Marks & Truscott, 1985). *Aster tripolium* viability was even lower at 6% overall, and only one of the naturally occurring seeds was viable. For perennial species, such as *S. anglica* and *S. maritimus*, a low viability is less of an issue due to rhizomatous and vegetative spread (Silinski et al., 2016; Zhu et al., 2014). For annual and biennial species, a low viability may be compensated by producing large amounts of seeds, to ascertain successful vegetation establishment.

Viability was highest for *S. procumbens*: between 80% and 90% at the surface layer. This was consistent for naturally occurring and supplied seeds (Figure 5 and Table 3). In the natural seed bank, viability dropped below 1 cm, except for the March treatment, where viability stayed above 50% up to 4 cm deep. A higher seed abundance with a higher viability below 1 cm found in the March treatment (Table 3 and Figure A1) is an indication that these seeds originated from the experimental seed addition. They have moved downwards into the soil, most likely through bioturbation (van Regteren et al., 2017). Natural *S. procumbens* seed abundance increased at a depth of 4–9 cm, while viability remained low (between 10% and 40% viability). This indicates that these seeds are older (Espinar et al., 2005), although most studies do not report on viability in relation to seed age (Wolters & Bakker, 2002). Viability of marsh seeds can decrease under anoxic conditions (Mossman, Brown, Davy, & Grant, 2012), and these conditions are quite common below the soil surface in the transition zone (personal observation). Ultimately, we can conclude that there was a seed bank present for *S. procumbens* in the transition zone and it contained a portion of viable seeds. This dormancy in *Salicornia* seeds may provide a survival tactic for less fruitful years (Davy et al., 2001).

### 4.4 Vegetation establishment and implications for management

Boundary conditions for *S. anglica* and *S. procumbens* establishment are appropriate at this location. In May, seedlings had established, with the highest cover in the March seed‐addition plots (Figure 5). *Aster tripolium* did not successfully establish (0%, Figure 5), most likely due to a combination of factors. Viability was low with 6% because it usually occurs in the lower marsh zone (Petersen et al., 2014) and the soil conditions in the transition zone may have been unfavorable (Erfanzadeh, Garbutt, et al., 2010). Although the number of seeds was not supplied evenly for the three species, the percentage of successful establishment was similar for *S. anglica* and *S. procumbens*. Total establishment success in the field was around 8% for *S. anglica* and 9% for *S. procumbens*. Despite *S. anglica* germination and establishment in the transition zone, natural *S. anglica* establishment from seeds is improbable, as no seed bank is present. Supplying *S. anglica* seeds in transition zones would likely lead to increased abundance in areas that cannot be reached clonally. Once established, *S. anglica* can reproduce vegetatively (through rhizomes) thereby inducing marsh succession, as seen in other marshes and with other perennials, typical for low and high marshes.

In the March seed‐addition plots, *S. procumbens* seedlings were abundant. They also showed natural establishment in the control and December treatment (Figure 5). The establishment mainly depended on seeds present in the surface layer. Only a few *S. procumbens* seeds were present in the surface layer of the control plots or December treatment but viability was high (Table 3). We suspect that those seeds naturally dispersed to our experimental plots after winter sediment erosion events took place. Possible adaptations for highly dynamic areas could be keeping seeds in dead stands until spring (observed in the field) inducing a prolonged dispersal period. Vegetation at the edge of the pioneer zone would benefit from a slow release of seeds. Additionally, seed viability was highest in more recent seeds, and thus, the greatest potential for expansion comes from seeds dispersed in the previous fall. The period from January to spring still forms a knowledge gap in terms of seed dispersal and retention for most marsh species. When aiming to stimulate initial vegetation establishment, further research on seed dispersal times and retention is essential for success.

Annual pioneers produce many seeds and are more dominant than perennials when colonizing new areas or the bare intertidal flat (Bossuyt & Honnay, 2008; Erfanzadeh, Petillon, Maelfait, & Hoffmann, 2010). For marsh expansion or marsh restoration, annuals will likely precede the establishment of perennials. Their initial establishment can aid subsequent marsh succession. Perennial pioneers, such as *S. anglica* and *S. maritimus*, are able to expand clonally onto the intertidal flat forming a slower but stable base. For longer distance dispersal and establishment, seeds are a vital component even for perennials (Zhu et al., 2014).

Salt marshes are excellent examples of nature‐based coastal defense (Temmerman et al., 2013), and their development can be actively promoted (Baptist, Gerkema, et al., 2019). We showed that seed availability can form a threshold for pioneer vegetation establishment, even with a source population in proximity. Successful management strategies aiming to promote salt‐marsh expansion should ensure the appropriate boundary conditions regarding seed availability, elevation, and sediment dynamics are met. They should take into account that natural seed availability may be reduced by secondary dispersal due to storms, particularly since higher storm frequencies are expected due to climate change (Woth, Weisse, & von Storch, 2006). More frequent storms, combined with expected sea‐level rise, will alter hydrodynamic regimes (Kirwan et al., 2010) and can thus further constrain lateral expansion and even decrease vegetated areas through cliff erosion (van der Wal & Pye, 2004). Seed availability is especially vital in highly dynamic transition zones, not only for salt marshes but also on the edges of mangroves or along vegetated riverbanks. If seed availability is a constraining factor, coastal management could consider, shortly before the establishment period, supplying seeds to strengthen natural barriers.

## 5 CONCLUSIONS

Overall, this study contributes to an advanced understanding of seed availability in the salt marsh–intertidal flat transition zone and how this can affect lateral expansion dynamics of salt marshes. Our results showed that (a) seed retention was successful with seed dispersal after winter, but unsuccessful with seed dispersal before winter due to sediment erosion during winter storms; (b) for *S. anglica* and *A. tripolium*, a natural viable seed bank was hardly present, and for *S. procumbens*, a seed bank existed up to 15 cm depth; (c) seeds from *S. anglica* and *A. tripolium* had low viability, respectively, 10% and 6%, whereas seeds from *S. procumbens* had a high viability, around 85% in the surface layer, and viability decreased with soil depth; and (d) windows of opportunity for vegetation establishment of *S. procumbens* and *S. anglica* were met in the field but not for *A. tripolium*. Low glasswort seed availability resulted in low seedling establishment for *S. procumbens*, and the absence of cordgrass seeds resulted in a lack of natural seedling establishment for *S. anglica*. However, for *A. tripolium* natural establishment was inhibited due to low viability of seeds and probably inappropriate boundary conditions. In transition areas where high dynamic sedimentary regimes are prone to occur, seed availability should be considered as a potential threshold for initial vegetation establishment.

## CONFLICT OF INTEREST

None declared.

## AUTHOR CONTRIBUTIONS

MR, MB, and KE conceived the ideas and designed methodology; MR, VF, MP, and IC collected the data; MR, PV, and IC analyzed the data; MR led the writing of the manuscript; and all authors contributed critically to revisions and gave final approval for publication.

## Data Availability

The final dataset used for statistical tests and model predictions is archived at 4TU, Datacentre http://researchdata.4tu.nl/home/, under doi number https://doi.org/10.4121/uuid:c877d345-7d6f-45ae-a442-21e07f5d1fa3.
