# Supplementary figures and images for: Limited seed retention during winter inhibits vegetation establishment in spring, affecting lateral marsh expansion capacity
Source: Ecol Evol. 2019 Nov 4;9(23):13294–308. doi: 10.1002/ece3.5781 (PMC6912888; doi:10.1002/ece3.5781)

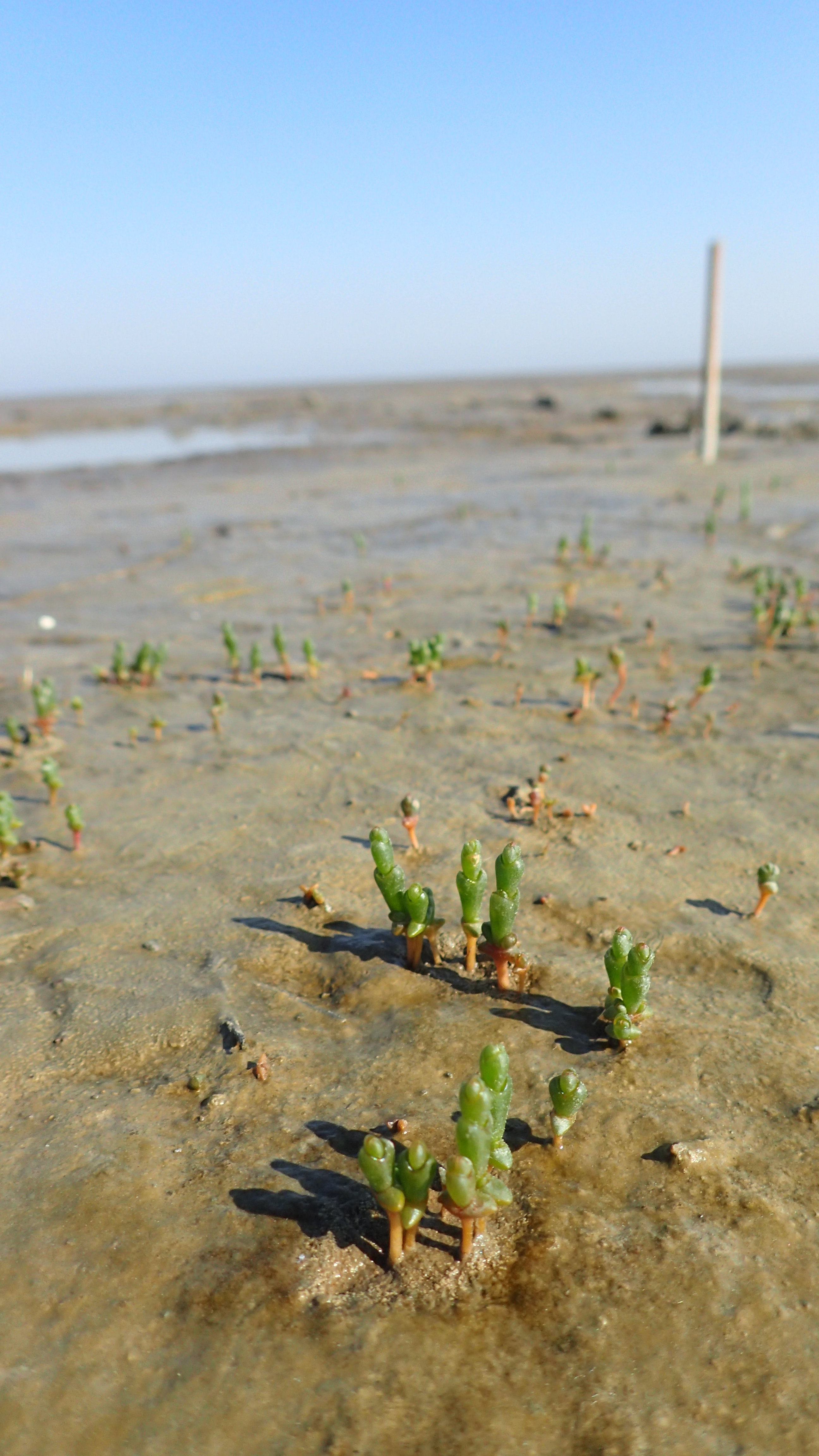

Supplement: Supplementary file 1 [file ECE3-9-13294-s001.jpg]
